# Delay in decision and determinants for safe abortion among women at health facilities in south West Ethiopia: facility based cross sectional study

**DOI:** 10.1186/s12939-020-1122-z

**Published:** 2020-01-07

**Authors:** Biru Abdissa Mizana, Tsige Woyecha, Samuel Abdu

**Affiliations:** 1 Department of Midwifery, Institute of Health, Jimma University, P.O.BOX: 378, Jimma, Ethiopia; 2 Jimma University Medical Center, Institute of Health, Jimma University, Jimma, Ethiopia; 3 School of Nursing and Midwifery, Institute of Health, Jimma University, Jimma, Ethiopia

**Keywords:** Delay in decision, Safe abortion, Southwest Ethiopia

## Abstract

**Background:**

Delayed safe abortion is the most common cause of gynecologic admission in developing countries. The study, therefore, assessed the delay decision for safe abortion and determinant factors among women at health facilities in South West Ethiopia.

**Methods:**

Facility-based cross-sectional study was conducted among 384 women who were selected from health facilities by using simple random sampling. A pre-tested structured questionnaire was used for data collection. Data were entered into Epidata and exported to SPSS for analysis. Binary Logistic regression was used and Variables with *P*-value < 0.25 during bivariate analysis were included in the multivariable logistic regression model. Finally, variables with *p*-value ≤0.05 were judged as a statistically significant association.

**Results:**

The magnitude of delay decision for safe abortion services was 70.8% (0.66, 075). Place of residence [AOR 2.44 (95% C.I: (1.39, 4.30)], lack of formal education [AOR: 2.41 (95% C.I:(1.08, 3.59)], level of education [AOR: 2.22 (95% C.I: (1.19, 4.11)], history of previous abortion [AOR: 3.47 (95% C.I: (1.74, 8.6.91)] and late confirmation of pregnancy [AOR: 1.64 (95% C.I: (1.01–2.65)] were the determinant factors for delay in decision for safe abortion.

**Conclusion:**

This study revealed that the majority of women were delayed for the decision of safe abortion services. Place of residence of the women, lack of formal education, history of previous abortion and late confirmation of pregnancy were the determinant factors for women’s decision for safe abortion. Therefore, it is better to work on awareness creation the timing of safe abortion and complication of delay abortion especially for the women from rural area.

## Background

Abortion is expulsion from the uterus of an embryo or fetus before viability of gestational age of fewer than 20 weeks or fetal weight less than 500 g [1]. According to Ethiopian abortion law, abortion is the termination of pregnancy before fetal viability, which is usually taken to be less than 28 weeks from last normal menstrual period (LNMP); and if the LNMP is not known a birth weight of less than 1000 g [2]. Safe abortion refers to a voluntary intervention to terminate an unwanted or unplanned pregnancy with skilled personnel in health facilities based on the countries abortion law [3]. Legal grounds on which abortion is permitted, according to revised abortion law of Ethiopia were when the pregnancy puts the woman’s life at risk, fetal impairment or gross deformity, when pregnancy is from rape or incest (based on the woman’s complaint only), when pregnancy occurs in minors (stated maternal age < 18 years), and when the woman is physically and mentally unable to care for coming, baby [4].

Ensuring that abortion care is safe, legal and accessible for all women is important as it decreases pregnancy-related deaths especially in unwanted pregnancy [5]. Achieving universal access ensures that wherever a woman seeks help, when she has unprotected sex, unwanted pregnancy, she will get care that she needs, whether it be information, referral or related services. This requires good political leadership, policy development, financial resources and an adequate health-system infrastructure with a trained health-care provider [6].

Delay for safe abortion will make abortion difficult as the fetal size increase, the complication wil increase. Evidence shows that suspecting pregnancy, confirming pregnancy, deciding to have an abortion, first asking for abortion and obtaining an abortion are the specific reasons for the delay [7]. Every day, about 800 women die from pregnancy and childbirth-related causes with majority death (99%) occurred in developing countries. Young adolescents face a higher risk of complications and death as a result of pregnancy than older women [8]. Ethiopia is among several low-income countries with the highest maternal morbidity and mortality in Sub-Sahara Africa. According to Ethiopia demographic and health survey 2016 report, maternal mortality ratio was 412 maternal deaths per 100,000 live births [9].

The severity of complications resulting from abortion can be reduced if appropriate abortion care is sought without delay. The effect of delays in providing care has long been accepted as the main determinant of maternal death [10]. Abortion accounts for about 13% of all maternal deaths globally and accounts as high as 50% of all maternal deaths in some Sub-Saharan Africa countries [11]. Delayed abortion is the most common cause of gynecologic admission based on data collected between 1988 and 2014 from 70 studies from 28 countries, it was estimated that at least 9% of abortion-related hospital admissions have a near-miss event and approximately 1.5% end in death [12]. In Kenya, among women attending post-abortion care, 46% of women presented with late abortions [13]. In Ethiopia, abortion-related complications contribute to about 31% of maternal mortality in the country [14].

A study conducted in England showed that 79% of respondents delayed in deciding to have an abortion [7]. Similarly, a study conducted by Queen’s University, Kingston researchers revealed that the prevalence of late presentation for abortion service was 70% [15]. Finding from the Amhara region referral hospital revealed that about 29.1% of women extend the decision to seek a safe abortion service beyond 10 days [16].

According to studies from Ethiopia and India, the age of women, marital status, level of education and place residence were among factors associated with delayed decision for safe abortion services [17–19]. Similarly, finding from Canada revealed that delay for abortion was because of discouragement from friends and family [15]. A cross-sectional study from California and China showed that delay to recognize pregnancy was a factor for the delay to decide for safe abortion services [19, 20]. A study conducted in Kenya revealed that the decision-making ability of women is a factor affecting delay decision to seek safe abortion service [21]. Government and non-government organization made an effort to decrease the death associated with unsafe abortion. Among these activities making laws of the countries to allow for safe termination of pregnancies where it is not against the law [22]. The study, therefore, assessed the delay in the decision for safe abortion and determinant factors among women at health facilities in South West Ethiopia.

## Methods

### Study design and setting

A facility-based cross-sectional study was conducted on women who came to health facilities for safe abortion services from April 1–28, 2019. The study conducted in Jimma town abortion clinics. Jimma town is one of the towns found in Jimma zone, Southwest Ethiopia which has 18 health facilities giving safe abortion services.

### Population and sampling

Study populations were all reproductive age group women who came to abortion clinics for safe abortion services during the study period. Those women with spontaneous/miscarriage or incomplete abortion and those who were seriously ill were excluded from the study. The sample size was determined by a single population proportion formula with the assumption of 50% prevalence, 5% marginal error, 95% confidence interval. Then, the final sample size was 384. Out of 18 health facilities giving safe abortion services in the study area, 8 health facilities were selected by simple random sampling (lottery method). The health facilities patients flow were checked by reviewing the six months of medical records before the study and based on this sample size was proportionally allocated for all eight health facilities. The study participants at each health facilities were selected by simple random sampling using lottery method by using the medical record number of the available patients on each day.

### Data collection procedure and data quality control

The structured questionnaire was prepared in English and translated to the regional language, Afaan Oromoo and translated back to English to check for consistency by language experts of both languages. The tools were prepared from different literature [6, 10, 13, 17, 20]. Reliability of the tools checked by Cronbach Alpha which was 0.839. The pre-test was done on 5% of total sample size and some modification was done on the tools. Data were collected from women by face- to- face interview on exit by six Bachelor degree holder female midwives and two master holder supervisors after giving training on study purpose and tools for two days. The principal investigators and supervisors checked the collected data daily for completeness.

### Measurement

Delay decision for safe abortion services was considered when the woman extends her decision of safe abortion beyond one week after she has confirmed her pregnancy in which the pregnancy was unwanted or unplanned and delay in confirming pregnancy was considered when women fail to diagnose or recognize pregnancy beyond five weeks after LNMP (last normal menstrual period). LNMP was used to confirm gestational age of her pregnancy but if she didn’t know her LNMP accurately we used early ultrasound result document. Pregnancy was confirmed by reviewing urine test or ultrasound examination result of the participants.

### Analysis

STROBE checklist was used to analyze and report data [13]. Data were entered into EpiData version 3.1 and exported to IBM SPSS version 23 for analysis. Both descriptive analysis like frequency and percentage and inferential statistics were done. Binary Logistic regression was employed. Odds ratio and *p*-value with a 95% confidence level were computed to judge the association between the outcome variable and independent variables. Those variables with a p-value less than 0.25 were entered into the final model. Those variables that had a p-value less than 0.05 after multivariable logistic regressions were considered as statistically significant association with outcome variables.

## Results

### Socio-demographic characteristics of respondents

A total of 384 women participated in the study which was 100% response rate. The mean (±SD) age of the women was 22.3 (± 4.4). Out of the total, 154 (40.1%) of respondents who obtained safe abortion services were at the age group of 20–24 years and more than half (57.8%) were single. More than one fourth (29.4%) of the respondents were at primary education and 183(47.7%) were Muslim religion followers (Table 1).

**Table 1:** Socio-demographic characteristics of participants in health facilities in South West Ethiopia, May 2019

Variables	Categories	Frequency	Percentage
Age	Less than 20	120	31.3
	20–24	154	40.1
	Above 24	110	28.6
Marital status	Single	222	57.8
	Married	123	32.0
	Divorced and separated	39	10.2
Residence	Urban	247	64.3
	Rural	137	35.7
Educational status	No formal education	58	15.1
	Primary education	113	29.4
	Secondary education	107	27.9
	Above secondary	106	27.6
Religion	Muslim	183	47.7
	Orthodox	116	30.2
	Protestants	79	20.6
	Others^a^	6	1.6
Ethnic background	Oromo	234	60.9
	Amara	56	14.6
	Dawuro	44	11.5
	Kaffa	23	6.0
	Tigre	9	2.3
	Others^b^	18	4.7
Occupation	Students	174	45.3
	House wife	84	21.9
	Unemployed	68	17.7
	Employed	52	13.5
	Others#	6	1.6

**Keys: -** ^a^ = Catholic and Wakefata ^b^ **=** Yem, Hadiya, Gurhaghe, Wolayita **#** = merchant, Daily laborers

### Reproductive characteristics of respondents

From the total, 119(31%) and 43 (11.2%) had a history of childbirth and previous abortion respectively. Twenty-six (6.8%) respondents had a history of multiple sexual partners. From the total, 355 (92.4%) respondents had an unwanted pregnancy and about half (52.9%) had a history of contraceptive use (Table 2).

**Table 2:** Reproductive characteristics of participants at health facilities in South West Ethiopia, May 2019

Variables	Categories	Frequency	Percentage
History of child birth (Parity)	Yes	119	31.0
	No	265	69.0
History of abortion	Yes	43	11.2
	No	341	88.8
History of multiple sexual partner	Yes	26	6.8
	No	358	93.2
Pregnancy status	Wanted	29	7.6
	Unwanted	355	92.4
History of contraceptive use	Yes	203	52.9
	No	181	47.1

### Delay in decision and the reason of delay for safe abortion

From the total, 272[(70.8%) (0.66,075)] had a delay in the decision for getting safe abortion services. Partner objection (40.4%), fear partner blame (69.9%), fear of abortion may not be safe (70%) and fear of pain from abortion procedure (73.2%) were mentioned as the reasons for delay for safe abortion.

### Determinants of delay in decision for safe abortion

After bivariate analysis, age group, residence, educational status, marital status, knowledge about free safe abortion service as safe abortion is free, history of previous abortion, being late to recognize pregnancy, and knowing presence of safe abortion had a *p*-value less than 0.25 and entered into multivariable logistic regression. After running multivariable logistic regression; residence, educational status, late recognition of pregnancy and history of previous abortion were determinants of delay decision for safe abortion.

Women’s residence was significantly associated with delay decision for safe abortion service. Women who came from rural were 2.44 times [AOR 2.44 (95% CI: 1.39–4.30)] more likely to be delayed to decide for safe abortion services than those who came from the urban area.

Educational status was among the factors associated with delay decision for safe abortion services. Women with no education were 2.41 times [AOR 2.41 (95% CI: 1.08–3.59)] more likely to be delayed to seek safe abortion service than women with education above the secondary school. Similarly, women with secondary educational level were 2.22 times [AOR 2.22 (95% CI: 1.19–4.11)] more likely to be delayed for safe abortion services than women with the educational level of secondary school and above.

History of previous abortion was one of the associated factors for delay decision for seeking safe abortion services. Women who had no history of previous abortion were 3.47 times [AOR 3.47 (95% CI: 1.74–8.6.91)] more likely to be delayed to seek safe abortion services than those who had no history of previous abortion. Those women who diagnosed their pregnancy late were 1.64 times [AOR 1.64 (95% CI: 1.01–2.65)] more likely to be delayed for safe abortion than women who have diagnosed their pregnancy early (Table 3).

**Table 3:** Determinant factors of delay in decision for safe abortion services at South West Ethiopia, May 2019

		Decision			
Variables	Categories	Delayed	Not Delayed	COR	AOR
Age group	Less than 20 Years	91(75.8)	29(24.2)	1.07 (0.59–1.95)	0.72(0.34–1.54)
	20–24 Years	99(64.3)	55(35.7)	0.62 (0.36–1.06)	0.64(0.34–1.22)
	Above 25 Years	82(75.5)	28(24.5)	1	1
Residence,	Urban	157(63.6)	90 (36.4)	1	1
	Rural	115(83.9)	22 (16.1)	2.99 (1.77–5.06)	2.44(1.39–4.30)*
Educational status,	No formal education	47(81)	11 (19)	3.28 (1.53–7.01)	2.41(1.08–5.39)*
	Primary education	83(73.5)	30 (26.5)	2.12 (1.20–3.74)	1.59(0.86–2.93)
	Secondary education	82(76.6)	25(23.4)	2.52 (1.39–4.53)	2.22(1.19–4.11)*
	Above secondary	60(56.6)	46(43.4)	1	1
Marital status	Single	155(69.8)	67(30.1)	0.51 (0.21–1.20)	0.50(0.19–1.27)
	Married	85(69.1)	38(30.9)	0.49 (0.19–1.21)	0.55(0.20–1.50)
	Divorced and Separated	32(82)	7(18)	1	1
Know presence of Legal Abortion	Yes	86(62.3)	52(37.7)	1	1
	No	186(75.6)	60(24.4)	1.87 (1.19–2.94)	1.34(0.79–2.25)
History of abortion	Yes	18(41.9)	25(58.1)	1	1
	No	254(74.5)	87(25.5)	4.05 (2.11–7.79)	3.47(1.74–6.91)*
Timely confirming pregnancy	Timely	92(61.3)	58(38.7)	1	1
	Late	180(76.9)	54(23.1)	2.10 (1.34–3.29)	1.64(1.01–2.65)*
Knowing price affordable (free)	Yes	157(75.1)	82(24.9)	1	1
	No	115(79.3)	30(20.7)	2.00(1.24–3.24)	1.38(0.79–2.41)

*p-value < 0.05

## Discussion

This study revealed that 70.8% of the respondents delayed in the decision of getting safe abortion services. This finding was lower than finding from England and Wales in which about 79% of respondents reported a delay in deciding to have a safe abortion [23]. This discrepancy may be due to the difference in study time and also might be due to differences in socioeconomic status. Also, this finding was higher than the study conducted in Amhara Region Referral Hospitals which showed 29.1% of women delayed in the decision to seek safe abortion service [24]. This difference might be due to the difference in study setting which means a study conducted in Amhara Region Referral Hospitals conducted only in government hospitals but the current study includes both governmental and private health facilities.

Women from the rural area were 2.44 times more likely to delay safe abortion services when compared with women from urban. Similarly, a study report from Canada Queensland and Bihar and Jharkhand, India revealed that rural women were more likely delayed for safe abortion service [17, 24]. This could be explained by the fact that those women from urban were more accessible to different social media and other sources of information concerning abortion and other related maternal health services and also women living in urban might have a good educational opportunity and due to these reasons women from urban might not be delay for safe abortion services.

The educational status of women was among the determinants of the decision of women for safe abortion services. Women with no education were 2.41 times more likely to be delayed for safe abortion service than those women with the educational status of above secondary school. Similarly, those women with secondary educational levels were 2.22 times more likely to be delayed to seek safe abortion services than those women with the educational status of above secondary school. This finding was in line with the study conducted in Bihar and Jharkhand, India which identified that less-educated women were more likely delayed to decide for safe abortion services than those women with higher educational levels [17]. This might be because women with no formal education cannot easily access information related to reproductive health and can’t easily understand the risk related to delayed abortion.

Early confirming pregnancy was also among the determinants affecting the delay of decision for safe abortion services. Those women diagnosed with their pregnancy late were 1.64 times more likely to be delayed to decide for safe abortion than those women who were diagnosed with their pregnancy early. This finding was supported by a study finding from Amhara Region Referral Hospitals which revealed those women who didn’t recognize their pregnancy early were more likely to delay safe abortion services than those women who did recognize their pregnancy early [24]. These might be explained by those women who recognize their pregnancy after their fetus grown might fail into a dilemma to continue or terminating their pregnancy and they may take a long time to decide for safe abortion.

Those women who had no previous history of abortion were 3.19 times more likely to be delayed for safe abortion services when compared with those women who had a history of previous abortion. This might be because women with a previous history of abortion were experienced with the process of safe abortion and they can easily decide to have safe abortion services.

Even though this study has much strength, it has limitations like social desirability bias and we didn’t consider the design effect because we gone through two stage of sampling which were selection of health facilities and selection of participants from selected health facilities. We didn’t identify the reason of delay for diagnosis of pregnancy in this study. Notwithstanding this limitation, we believe that our study has very important findings in the study area and areas with similar set up to fight maternal death related to abortion.

## Conclusion

The majority of women did not decide timely to get a safe abortion service which is critical for the health of women. The study also identified; place of residence, educational status, timely confirmation of pregnancy and previous history of abortion were determinants of delay decision for safe abortion. Hence, it is vital to work on awareness creation for rural women concerning the timing of safe abortion and complication of delayed abortion. We would like to recommend researcher to study about psycho-social complications of abortion and reason of delay for diagnosis of pregnancy.

## Data Availability

The datasets used during the current study were available from the corresponding author on reasonable request.

## Abbreviations

AOR: Adjusted odd ratio
BSc: Bachelor of Science
CI: Confidence Interval
COR: Crude Odd Ratio
LNMP: Last Normal Menstrual Period
MSc: Master of Science
SPSS: Statistical Package for Social Science
